# Coupling of Single Molecule, Long Read Sequencing with IMGT/HighV-QUEST Analysis Expedites Identification of SIV gp140-Specific Antibodies from scFv Phage Display Libraries

**DOI:** 10.3389/fimmu.2018.00329

**Published:** 2018-03-01

**Authors:** Seung Yub Han, Alesia Antoine, David Howard, Bryant Chang, Woo Sung Chang, Matthew Slein, Gintaras Deikus, Sofia Kossida, Patrice Duroux, Marie-Paule Lefranc, Robert P. Sebra, Melissa L. Smith, Ismael Ben F. Fofana

**Affiliations:** ^1^Biology Department, Boston College, Chestnut Hill, MA, United States; ^2^Department of Genetics and Genomic Sciences, Icahn School of Medicine at Mount Sinai, Icahn Institute of Genomics and Multiscale Biology, New York, NY, United States; ^3^The international ImMunoGeneTics information system^®^ (IMGT^®^), Laboratoire d'ImmunoGénétique Moléculaire (LIGM), Institut de Génétique Humaine (IGH), UMR CNRS, Montpellier University, Montpellier, France

**Keywords:** antibody, simian immunodeficiency virus, rhesus macaque, PacBio sequencing, single chain fragment variable library, phage display, International ImMunoGeneTics information system/HighV-QUEST

## Abstract

The simian immunodeficiency virus (SIV)/macaque model of human immunodeficiency virus (HIV)/acquired immunodeficiency syndrome pathogenesis is critical for furthering our understanding of the role of antibody responses in the prevention of HIV infection, and will only increase in importance as macaque immunoglobulin (IG) gene databases are expanded. We have previously reported the construction of a phage display library from a SIV-infected rhesus macaque (*Macaca mulatta*) using oligonucleotide primers based on human IG gene sequences. Our previous screening relied on Sanger sequencing, which was inefficient and generated only a few dozen sequences. Here, we re-analyzed this library using single molecule, real-time (SMRT) sequencing on the Pacific Biosciences (PacBio) platform to generate thousands of highly accurate circular consensus sequencing (CCS) reads corresponding to full length single chain fragment variable. CCS data were then analyzed through the international ImMunoGeneTics information system^®^ (IMGT^®^)/HighV-QUEST (www.imgt.org) to identify variable genes and perform statistical analyses. Overall the library was very diverse, with 2,569 different IMGT clonotypes called for the 5,238 IGHV sequences assigned to an IMGT clonotype. Within the library, SIV-specific antibodies represented a relatively limited number of clones, with only 135 different IMGT clonotypes called from 4,594 IGHV-assigned sequences. Our data did confirm that the IGHV4 and IGHV3 gene usage was the most abundant within the rhesus antibodies screened, and that these genes were even more enriched among SIV gp140-specific antibodies. Although a broad range of VH CDR3 amino acid (AA) lengths was observed in the unpanned library, the vast majority of SIV gp140-specific antibodies demonstrated a more uniform VH CDR3 length (20 AA). This uniformity was far less apparent when VH CDR3 were classified according to their clonotype (range: 9–25 AA), which we believe is more relevant for specific antibody identification. Only 174 IGKV and 588 IGLV clonotypes were identified within the VL sequences associated with SIV gp140-specific VH. Together, these data strongly suggest that the combination of SMRT sequencing with the IMGT/HighV-QUEST querying tool will facilitate and expedite our understanding of polyclonal antibody responses during SIV infection and may serve to rapidly expand the known scope of macaque V genes utilized during these responses.

## Introduction

Nonhuman primates are an important animal model for numerous human diseases, as there is great similarity between the human and macaque genomes (1–7). In addition, macaque immunoglobulin (IG) genes are likely those most closely related to human IG genes among available human immunodeficiency virus (HIV)/acquired immunodeficiency syndrome (AIDS) animal models (8–11). As a result, the variable heavy (VH) and variable light (VL) domains of macaque antibody heavy (H) and light (L) chains can be generated using polymerase chain reaction (PCR) conditions and oligonucleotide primers based on human IG nucleotide sequences. This has been shown thus far for VH rather than VL genes, although the use of rhesus specific primers for amplification may function for both variable domains (9, 11, 12).

The simian immunodeficiency virus (SIV)/macaque model of AIDS has been extensively studied and provides the most accurate reflection of HIV pathogenesis across all available animal models (5, 13–16). Virus-specific antibodies are abundantly produced during the course of HIV/SIV infection in humans or macaques (5, 17–20). These antibodies, which primarily target the envelope glycoprotein (Env) on the surface of HIV/SIV virions, generally do not provide protection due in part to their inability to neutralize the virus. In fact, the Env surface glycoprotein employs multiple strategies to shield its neutralization-sensitive epitopes, such as the CD4 and the coreceptor-binding sites, as well as the fusion peptide on the Membrane Proximal External Region (MPER). Env trimer oligomerization, the presence of hypervariable loops and extensive glycosylation are all components of a complex escape mechanism that limits the potential potency of antibody-mediated neutralization (5, 21).

Despite this, many potent HIV neutralizing antibodies have been isolated and characterized (22–26). Unique structural features of these antibodies, including extensive somatic mutations and unusually long VH domain complementarity determining region three (VH CDR3), have been associated with the development of potent neutralizing activity (19). However, roles for the VL domains and other complementarity determining regions in conferring potent HIV neutralizing activity have not been excluded (27–29).

Despite the extensive knowledge gained from studying this panel of naturally occurring neutralizing antibodies, stimulating the development of broadly neutralizing antibodies (bnAbs) by vaccination has remained a critical roadblock in the development of an efficacious HIV vaccine. The failures of past vaccine immunogens to do so have been, in part, attributed to the inability of the various vaccine antigens to engage B cells that express the proper germline receptors (30–33). However, progress in this area is being made. For example, very recently anti-HIV bnAbs were elicited in the cow (*Bos taurus*), an unusual experimental animal for HIV-related research (34). The authors were motivated to take this approach due to the inherently long VH CDR3, which characterize cow antibodies. The rarity of such long VH CDR3 in the human IG repertoire raises the question of whether induction of such long VH CDR3 bnAbs will ever be achieved in humans *via* vaccination. In light of these questions and challenges, many have also turned their focus to the possibility of eliciting protective non-neutralizing antibodies (nnAbs), which may also be capable of preventing HIV infection. Interestingly, the most successful clinical HIV vaccine trial to date, RV144, found a correlation with limited protection and non-neutralizing Env-binding antibodies that target the V1-V2 loop (35–38). Similarly, the most successful pre-clinical HIV vaccine, which uses live-attenuated strains of SIVmac239 found only low titers of neutralizing antibodies in protected macaques; suggesting instead a role for nnAbs in this model (39). Unlike nAb activity, which appears to be conferred by a single or few dominant, protective monoclonal antibodies (mAbs) in a given individual; the characterization of protective nnAbs will likely require large-scale analysis of polyclonal antibodies.

We have previously reported the construction of a phage display library from a SIV-infected rhesus macaque (11). This library was generated by PCR amplification of the VH and VL chains using primers corresponding to the human IG gene sequences. Our prior screening of mAbs from this library relied on handpicking bacterial clones after biopanning with SIV Env gp140, followed by Sanger sequencing. This approach was inefficient and generated only a few dozen sequences for analysis, likely severely underrepresenting the repertoire present. The screening of this library would be greatly improved with the use of next generation sequencing (NGS) technologies, such as Illumina, however often NGS platforms are limited in the length of the reads (≤400 bp), covering at most one VH or VL domain per read. This limitation has been addressed through the use of alternate high throughput NGS platforms such as the Ion Torrent Personal Genome Machine (PGM) S5 and the Pacific Bioscience (PacBio) RSII and Sequel systems (40, 41). Using the PGM-S5 system, He et al. generated 900 bp sequencing reads to identify precursors and lineage intermediates of HIV-1 bnAbs from a phage display library. Although single chain fragment variables (scFvs) from this combinatorial library did not necessarily represent authentic VH–VL pairing, the authors were able to validate their data using biopanning onto a native-like gp140 trimer and comparison with previously characterized bnAb lineages (42–49). Using the PacBio RSII system, Hemadou et al. generated long reads (>800 bp) covering full length scFvs following *in vivo* panning in an animal model of atherosclerosis. Subsequently, they analyzed the sequencing data using International ImMunoGeneTics information system (IMGT)/HighV-QUEST combined with a novel scFv functionality tool for simultaneous characterization of VH and VL chains from individual scFvs. Here, using our previously characterized phage display library (11), we tested the validity of PacBio sequencing and IMGT/HighV-QUEST analysis (www.imgt.org) combined with scFv functionality for the identification and characterization of SIV-specific antibodies.

## Materials and Methods

### Phage Display Library Preparation

Construction of a scFv phage display library using archived spleen biopsies from a SIV-infected rhesus macaque (Mm333-95) has been previously reported (11, 50). The animal was housed at the New England Primate Research Center of Harvard Medical School, and given care in accordance with standards of the Association for Assessment and Accreditation of Laboratory Animal Care and the Harvard Medical School Animal Care and Use Committee. The study was approved by the Harvard Medical Area Standing Committee on Animals, within the Office for Research Subject Protection at Harvard Medical School, and conducted according to the principles described in the *Guide for the Care and Use of Laboratory Animals* (51).

In the current study, we aimed to evaluate our sequencing and analysis pipelines for the characterization of SIV-specific antibodies. For that reason, the previously generated library was used (11) as it allowed for the most direct comparison of the new pipelines with the prior gold standard method of handpicking colonies representing selected SIV-specific scFvs and screening by Sanger sequencing.

In the first round, antibody variable domains, VH and VL, were amplified by PCR using oligonucleotide primers corresponding to the human IG sequences. In the second round, VH and VL products were linked together using external primers corresponding to the 5′ (RSC-F: gaggaggaggaggaggaggcggggcccaggcggccgagctc) and 3′ (RSC-B: gaggaggaggaggaggagcctggccggcctggccactagtg) regions of the VL and VH PCR products, respectively. This fusion was facilitated by the addition of a linker sequence to the internal PCR primers corresponding to 3′ and 5′ regions in VL and VH PCR products, respectively. The resultant scFv (VL-linker-VH) products were cloned into the phagemid vector pComb3xSS. XL1 Blue *Escherichia coli* were transformed with recombinant pComb3xSS-scFv DNA by electroporation using a Gene Pulser Xcell (Bio-Rad, Hercules, CA, USA). The phage library preparation was obtained after amplification by culturing in the presence of VCSM13 helper phage (Agilent, Santa Clara, CA, USA). Biopanning of the library using immobilized SIV Env gp140 was also previously described (11).

### scFv DNA Preparation from Library and Sub-Library

Total DNA was extracted from the bacterial pellets obtained during preparation of the unpanned library and the fourth round SIV Env gp140-panned library using the PureLink DNA maxi-preparation kit (Invitrogen, Carlsbad, CA, USA). scFv DNA was then PCR amplified from the extracted DNA using the same external primers (RSC-F and RSC-B) that had been selected for initial library construction. Samples were amplified in triplicate, with three different concentrations (25, 50, and 75 ng) of DNA as initial template input. High-fidelity Platinum SuperFi polymerase (Life Technologies, Carlsbad, CA, USA) was used to prepare the reaction mixture as follows: 1 µl template DNA (25, 50, or 75 ng), 2 µl (200 pmol) 5′ Primer (RSC-F), 2 µl (200 pmol) 3′ Primer (RSC-B), and 45 µl Platinum SuperFi Master mix. PCR reactions were performed under the following conditions: heated to 94°C for 5 min, subjected to 15 cycles of: 94°C for 15 s, 56°C for 15 s, 72°C for 2 min, followed by a 10-min extension at 72°C. Five microliters of each reaction were evaluated for successful amplification on a 1% agarose gel. The triplicate reactions were then pooled together before performing a PCR cleanup using the QIAquick PCR purification Kit (Qiagen, Valencia, CA, USA).

### SMRTbell Library Preparation and Sequencing

PacBio SMRTbell library preparation and sequencing has previously been described (52). Briefly, SMRTbell libraries were prepared following the manufacturer’s protocol and using the SMRTbell Template Prep Kit 1.0 (Pacific Biosciences, Menlo Park, CA, USA). A total of 250 ng of AMPure PB bead-purified scFv amplicon was added directly to the DNA damage repair step of the Amplicon Template Preparation and Sequencing protocol (http://www.pacb.com/wp-content/uploads/2015/09/Unsupported-Amplicon-Template-Preparation-Sequencing.pdf). Following construction, SMRTbell library quality and quantity were assessed using both the Agilent 12000 DNA Kit and the 2100 Bioanalyzer System (Santa Clara, CA, USA), as well as the Qubit dsDNA High Sensitivity Assay kit and Qubit Fluorometer (Thermo Fisher Scientific, Waltham, MA, USA). Sequencing primer annealing and P6 polymerase binding were performed using the recommended 20:1 primer:template ratio and 10:1 polymerase:template ratio, respectively. scFv SMRTbell libraries were loaded onto SMRT cells at a concentration of 50 pM. SMRT sequencing was performed on the PacBio RS II system using the C4 sequencing kit with magnetic bead loading and 6-h movies. Circular consensus sequencing (CCS) reads were generated using Arrow and the CCS2 protocol as a part of SMRTLink version 4.0; CCS reads were filtered by both quality (Q30, 99.9% accuracy) and size, retaining only sequences that ranged from 700 to 1,200 bp based on expected scFv size distribution. Resultant FASTA files were used for downstream analyses. These filtered CCS reads have been submitted to the Sequence Read Archive under submission SUB3223332 entitled “*Macaca mulatta*-derived SIV-gp140-specific scFv circular consensus sequences” and can be retrieved using accession number SRP125114.

### IMGT/HighV-QUEST Analysis

International ImMunoGeneTics information system/HighV-QUEST analysis was performed *via* the IMGT web portal (53–57). The CCS FASTA files were analyzed using IMGT/HighV-QUEST program version 1.5.5 with the advanced scFb functionality (57). Resultant data files obtained from IMGT/HighV-QUEST were further analyzed using the statistical and clonotype analysis tool that uses the IMGT/V-QUEST version 3.4.7 with advanced scFv functionality (57). An IMGT clonotype (AA) (55) is defined as a unique V-(D)-J rearrangement (with the IMGT gene and allele names determined by IMGT/HighV-QUEST at the nucleotide level) and a unique CDR3-IMGT AA in-frame junction sequence (C104, W118 for IGHV and C104, F118 for IGKV and IGLV) (see IMGT/HighV-QUEST documentation). Data filtering was applied with the following criteria to be fulfilled for each of the two V domains: (i) >85% of identity of the V-REGION of the V domain with the V-REGION of the closest germline IMGT gene and allele (58) and (ii) in-frame V-(D)-J junction. Filtered sequences were then analyzed to identify the closest V, D (for VH) and J IMGT genes and alleles, in order to characterize the amino acid (AA) junction and to give a complete description of the scFv with IMGT labels, using the IMGT/V-QUEST algorithm for scFv, implemented in IMGT/HighV-QUEST. Here, we report the statistical and IMGT clonotype analysis for each domain for the unpanned library (Pan0) and the fourth round SIV Env gp140 panned library (Pan4). In order to ensure that the breadth and depth of the data generated by a single SMRT cell per sample was not limiting the characterization of the complex scFv pools, a comparative analysis was done pooling data generated from three SMRT cells run for a single sample.

### scFv Recovery from the Sub-Library

Two clones were selected from the fourth round of gp140-panning based on extent of VH CDR3 representation among the sequences generated by IMGT analysis. In addition, these clones also contained a new VH CDR3 AA sequence compared to previously identified clones (11). Recovery of scFv from libraries has previously been described (59). Briefly, overlapping primers were design within the VH CDR3. For clone selection one (S1), the 5′ primer (S1-F: GCG AGA GGC TCC AAA CAA TTT TGT AGT) and 3′ primer (S1-R: ACT ACA AAA TTG TTT GGA GCC TCT CGC) were used while for clone selection two (S2), the 5′ primer (S2-F: CCT CTC CCC GAC TGG GCT GAT TAT AAG) and 3′ primer (S2-R: CTT ATA ATC AGC CCA GTC GGG GAG AGG) were selected. PCR was conducted as described above, using the Platinum HiFi Mastermix (Life Technologies, Carlsbad, CA, USA). 1 µl of DpnI digested product was used to transform TOP10 F′ *E. coli* (Thermo Fisher Scientific, Waltham, MA, USA) and the subsequently transformed bacteria were plated on LB/Agar medium supplemented with 50 µg/ml of Carbenicillin (AmericanBio, Natick, MA, USA). Positive clones were verified by Sanger sequencing. The recovered sequence was also analyzed using IMGT/V-QUEST(www.imgt.org) with scFv functionality, as described above.

Following this selective cloning, binding specificity to SIV Env gp140 of these clones were confirmed after induction of the bacterial culture as previously described (8, 11, 20). Briefly, bacterial colonies were cultured with SB medium supplemented with 50 µg/ml of Carbenicillin (AmericanBio, Natick, MA, USA). After 5–8 h of culture at 37°C and 250 rpm, scFv expression was induced by addition of Isopropyl β-D-1-thiogalactopyranoside (IPTG) at 2 mM. The induction was performed at 37°C overnight. Culture supernatant was clarified by centrifugation at 12,000 rpm in a microcentrifuge for 5 min. 50 µl of supernatant was tested by enzyme-linked immunosorbent assay (ELISA) for binding to SIV Env gp120 and/or gp140. ELISA plates were also coated with BSA as a negative control antigen.

## Results

### SMRT Sequencing

**Figure 1:**
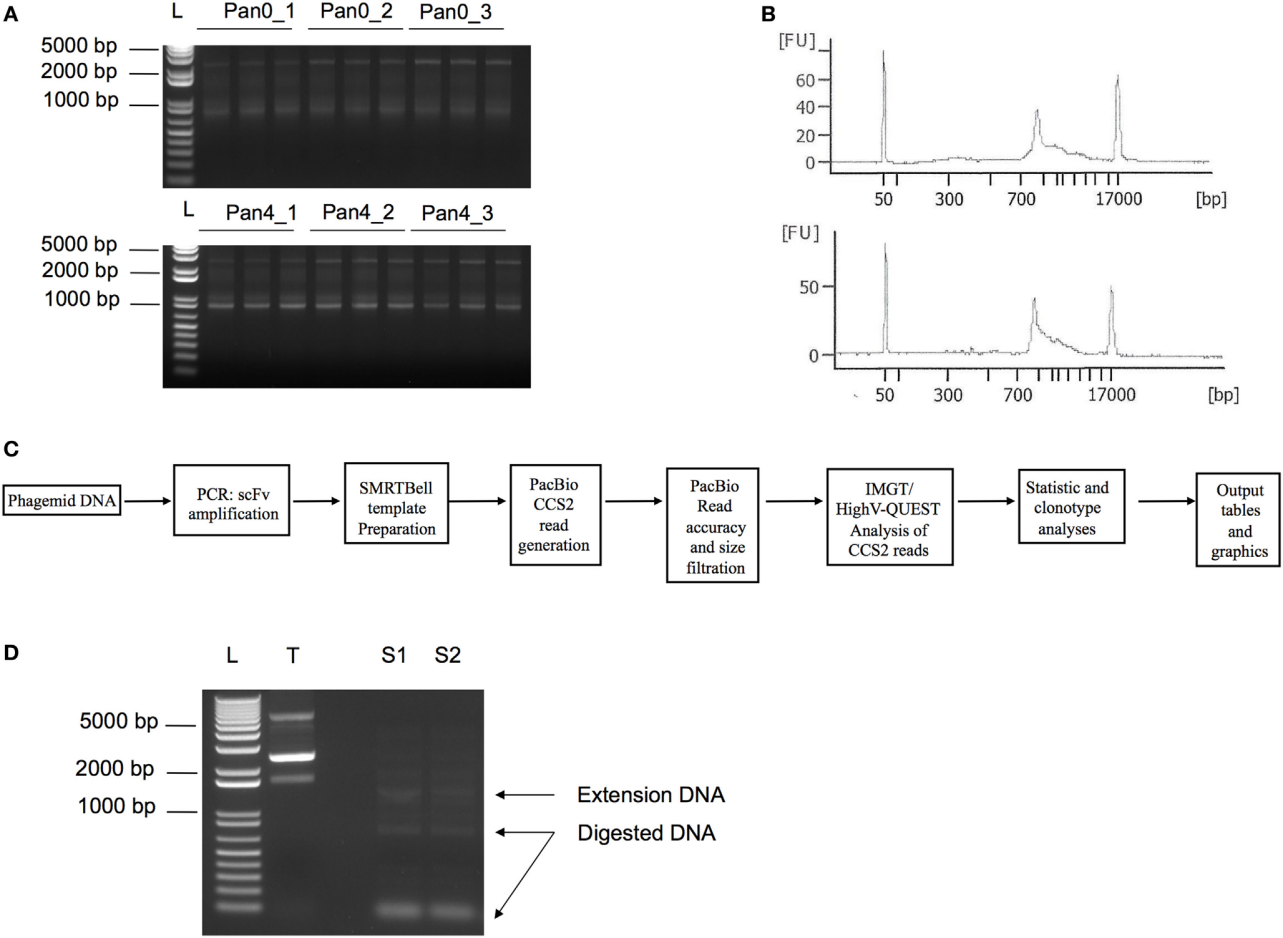
PCR amplification of scFv libraries. scFv DNA was amplified using human IG-specific PCR primers. **(A)** Triplicate amplified samples were generated using 25, 50, and 75 ng of input DNA template for Pan0_1, Pan0_2, or Pan0_3 and Pan4_1, Pan4_2, or Pan4_3, respectively. Pan0 correspond to the unpanned library while Pan4 represents fourth round selection onto gp140. **(B)** DNA traces generated using the DNA12000 kit on the Agilent Bioanalyzer and represent the distribution of scFv amplicons based on size. **(C)** Key steps in SMRT sequencing, IMGT/HighV-QUEST and clonotype analysis. **(D)** PCR amplification of selected scFv clones chosen for functional validation, S1 and S2, followed by Dpn1 digestion. T, control non-amplified and non-digested DNA; L, 1 Kb plus DNA ladder; bp, base pair; FU, florescence units; scFv, single chain fragment variable; SMRT, single molecule, real-time.

We have previously described the construction of a phage display library derived from a rhesus macaque infected with SIV. This work generated 32 unique scFv sequences after four rounds of panning onto SIV Env gp140, all of which were characterized to target the gp41 region of Env (11). Here, we aimed to perform a large-scale deep sequencing-driven analysis of the same scFv library and compare the diversity of the unpanned library (Pan0) to the library after round 4 of gp140 selection (Pan4). DNA extracted from bacterial pellets obtained after Pan0 and Pan4 culturing were used as templates to amplify the collection of scFv sequences under examination (Figure 1).

As expected, we obtained DNA amplicons of ~800 bp representing the variable domains VH (~ 400 bp), VL (~350 bp) and the short (21 bp) or long linker (54 bp) (Figure 1). We also obtained additional and less pronounced products between 800 and 1,700 bp (Figures 1A,B) with the 1,700 bp fragment corresponding to scFv dimers. Furthermore, peaks at 50 and 17,000 bp were also observed and corresponded to the lower and upper ladder of the Agilent 12000 DNA Kit, respectively (Figure 1B). In order to focus our analyses on highly accurate, full-length scFv fragments, SMRT sequencing data were processed using the latest CCS2 algorithm and filtered by size (700–1,200 bp) and accuracy (99.9%, QV30) (Figure 1C). Table 1 summarizes the total number of sequences and IMGT clonotype-respective assignments of the VH and VL domains to the IGHV, IGKV, and IGLV genes observed. The total number of sequences assigned to an IMGT clonotype was independent of PCR template DNA concentration and similar between Pan0 (3,837, 3,159, and 5,238) and Pan4 (4,594, 4,594, and 3,884). As would be expected following antigen-specific selection, the analysis of IGHV sequences resulted in many more distinct clonotypes in Pan0 (1,887, 1,685, and 2,569) than Pan4 (135, 141, and 127). The number of observed out-of-frame sequences was similar between Pan0 (117, 90, and 115)and Pan4 (106, 115, and 80). The number of “other category” sequences was far higher for Pan0 (112, 76, and 103) than Pan4 (1, 0, and 0) suggesting that the Pan4 products contain more productive sequences than were present in the unpanned library. A total of 4,640 (Pan0) and 6,957 (Pan4) IGKV sequences and a total of 4,068 (Pan0) and 7,484 (Pan4) IGLV sequences were assigned to an IMGT clonotype. A more diverse pool of clonotypes was observed with Pan 0, consisting of 1,651 IGKV and 1,573 IGLV variants. The Pan4 sublibrary was far less diverse with only 147 and 588 different clonotypes for IGKV and IGLV, respectively.

**Table 1:** IGHV, IGKV, and IGLV genes, sequence number and IMGT clonotype (AA) assignment.

	Pan0_1	Pan0_2	Pan0_3	BatchPan0	Pan4_1	Pan4_2	Pan4_3	BatchPan4
PacBio Output seq Nb	8,706	7,335	11,599	27,640	15,036	14,986	13,231	43,253
Nb of seq assigned to an IMGT clonotype (AA) for IGHV	3,837	3,159	5,238	12,269	4,594	4,593	3,884	13,087
Nb of different IMGT clonotypes (AA) for IGHV	1,887	1,685	2,569	5,313	135	141	127	247
Nb of out-of-frame seq for IGHV	117	90	115	322	106	115	80	301
Nb of seq of other categories for IGHV	112	76	103	292	1	0	0	1
Nb of seq assigned to an IMGT clonotype (AA) for IGKV	3,511	2,818	4,640	11,205	6,957	7,062	5,974	19,995
Nb of different IMGT clonotypes (AA) for IGKV	1,280	1,099	1,651	3,263	147	168	150	280
Nb of out-of-frame seq for IGKV	126	96	172	409	216	248	214	678
Nb of seq of other categories for IGKV	54	29	50	137	2	6	3	11
Nb of seq assigned to an IMGT clonotype (AA) for IGLV	3,110	2,531	4,068	9,910	7,484	7,252	6,504	21,502
Nb of different IMGT clonotypes (AA) for IGLV	1,268	1,077	1,573	3,205	588	548	535	1,078
Nb of out-of-frame seq for IGLV	209	169	235	641	581	570	488	1,639
Nb of seq of other categories for IGLV	67	60	111	240	18	22	27	67

*Filtered CCS reads (≥99.9% accuracy and 700–1,200 bp in length) generated from SMRT sequencing data were analyzed using IMGT/HighV-QUEST in order to determine V, D, J gene sequence, allele assignment and functionality (53, 54). IMGT/HighV-QUEST output files were processed through IMGT statistical and clonotype analysis to determine the number of assigned sequences and clonotypes. The number of sequences assigned to an IMGT clonotype corresponds to in-frame sequences [C104, W118 for VH and C104, F118 for VL (V-KAPPA or V-LAMBDA)] from “1 copy” + “More than 1,” “Single allele” and “Several alleles” (or genes) (41). Number of in-frame “other categories” sequences were not assigned to a “single gene.” An IMGT clonotype (AA) was defined by a unique V-(D)-J rearrangement (with IMGT gene and allele names determined by IMGT/HighV-QUEST at the nucleotide level) and a unique CDR3-IMGT AA in-frame junction sequence (55, 56). Pan0 correspond to the unpanned library while Pan4 represent fourth round of selection onto SIV Env gp140. Batch0 and Batch4 represent the analysis of pooled data from three independent SMRT cells for Pan0 and Pan4, respectively. Nb, number; seq, sequence. PCR template concentration of 25, 50, and 75 ng are represented by Pan0_1, Pan0_2, and Pan0_3 or Pan4_1, Pan4_2, and Pan4_3, respectively*.

### scFv Recovery

A manual inspection of the VH CDR3 AA sequences in the newly assigned clonotypes allowed for the verification of the presence of clonotypes previously identified by handpicking and Sanger screening of clones obtained from Pan4 (11). As more than 100 additional clonotypes were observed, the validity of the PacBio-generated sequences was verified by testing the SIV-specificity of two newly identified clones belonging to two different IMGT IGHV clonotypes. Of the selected (S1 and S2) clonotypes, S1 IGHV was not abundantly represented in the pooled data, with only 3 sequences observed, while S2 IGHV was represented at a higher proportion, with 102 sequences. Two S1 and S2 clones were recovered by PCR using DNA Pan4 as the template (Figure 1D). The identities of the recovered clones were confirmed by Sanger sequencing and the clonotype characteristics were analyzed using IMGT/V-QUEST with scFv functionality (Table 2). Somatic hypermutation (SHM) frequencies were determined using nucleotide identity with the closest germline as obtained with IMGT/V-QUEST. To confirm functional activity, the recovered clones were evaluated by ELISA to characterize binding to SIV Env (Table 2).

**Table 2:** Characteristic of two scFv clones recovered from Pan4.

scFv	S1	S2
IGHV gene and allele	IGHV1-1*01 F	IGHV3-5*01 F
IGHV SHM (%)	20.7	7.14
VH CDR3 AA sequence	ARGSKQFCSSSYCSVGFDY	AAEPLPDDWADWADYKKYGLDY
IGLV gene and allele	IGLV2S1*01 F	IGLV1-10*01 F
IGLV SHM (%)	5.65	6.07
V-LAMBDA CDR3 AA sequence	SSYAGSNTFLF	AAWDDSLSGWIF
Target	gp41	gp41

*V genes and alleles, and complementarity determining regions 3 (VH CDR3 and V-LAMBDA CDR3) were determined using IMGT/V-QUEST with scFv functionality (57). Somatic hypermutation frequencies [SHM (%)] represent the nucleotide sequence divergence from the closest germline in IMGT/V-QUEST (54). scFv binding specificity was determined by enzyme-linked immunosorbent assay (ELISA)*.

*F, functional; AA, amino acid*.

### V-D-J Assignments

Data files derived from the IMGT/HighV-QUEST analysis of the PacBio CCS2 reads were submitted to the statistical and IMGT clonotype analysis using the new scFv functionality tool on the IMGT/HighV-QUEST web portal (45) (www.imgt.org). The sequence analysis pipeline is provided in Figure 1C.

#### IGHV

**Figure 2:**
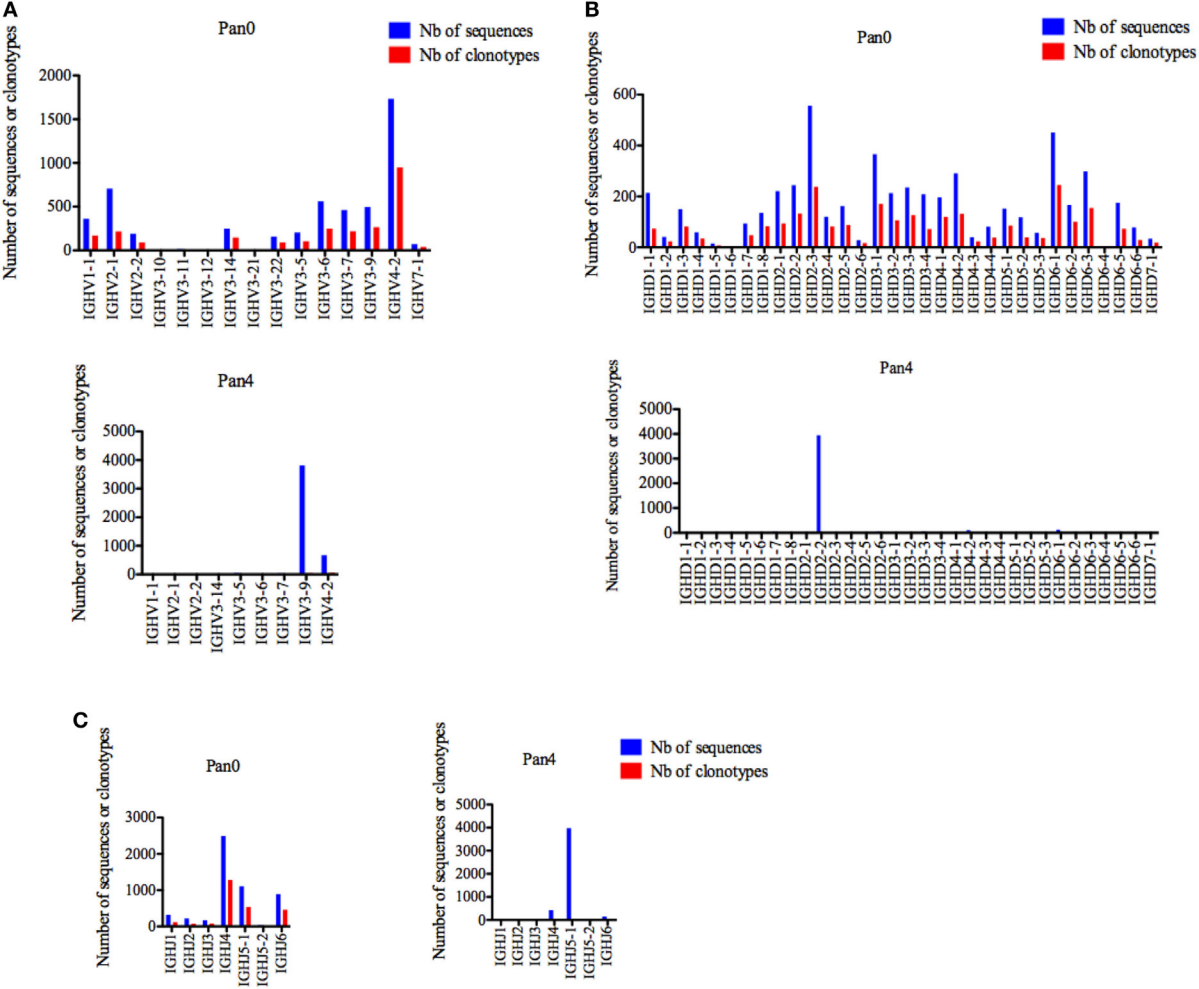
Number of IGH sequences and clonotypes, per IGHV, IGHD, and IGHJ gene, in Pan0 and Pan4 single chain fragment variable libraries. A International ImMunoGeneTics information system (IMGT) clonotype [amino acid (AA)] was defined as a unique V-(D)-J rearrangement using IMGT gene and allele names as determined by IMGT/HighV-QUEST at the nucleotide level, and a unique, in frame variable heavy CDR3-IMGT AA junction (C104, W118) (56). **(A)** IGHV; **(B)** IGHD; **(C)** IGHJ. Nb: number.

Pan0, which reflects a broad representation of total antibody genes in this macaque, was highly diverse with a single gene (IGHV1-1, IGHV4-2, and IGHV7-1) representing the IGHV1, IGHV4, and IGHV7 subgroups, two genes (IGHV2-1 and IGHV2-2) for the IGHV2 subgroup and 10 genes (IGHV3-5, IGHV3-6, IGHV3-7, IGHV3-9, IGHV3-10, IGHV3-11, IGHV3-12, IGHV3-14, IGHV3-21, and IGHV3-22) for IGHV3 (Figure 2A). Individually, IGHV4-2 was the most represented gene in the Pan0 dataset, with 1,734 (33.14%) assigned sequences. From a subgroup perspective, IGHV3 was the most represented with a total of 2,165 (41.31%) assigned sequences. Within IGHV3, three genes, IGHV3-6, IGHV3-9, and IGHV3-7, were the most abundant with 562, 498, and 462 assigned sequences, respectively. Overall, the number of assigned IMGT clonotypes was proportional to the number of sequences, as observed for IGHV4-2 (36.97%), IGHV3-6 (9.71%), IGHV3-9 (10.37%), and IGHV3-7 (8.54%).

For Pan4, a total of 9 IGHV genes were observed, compared to 14 in Pan0. IGHV3 remained the most represented subgroup with 6 genes (IGHV3-5, IGHV3-6, IGHV3-7, IGH3-9, and IGHV3-14), followed by IGHV2 with 2 genes (IGHV2-1 and IGHV2-2) and IGHV1 and IGHV4 with a single gene each (IGHV1-1, IGHV4-2) identified. The ranking in allelic representation was generally similar, with the striking exception of a switch between the 2 most represented genes, including 3,814 (83.05%) sequences for IGHV3-9 and 671 (14.61%) sequences for IGHV4-2. IGHV3-6 and IGHV3-7, which were well represented in Pan0, were reduced to only 2 (0.04%) and 35 (0.76%) sequences, respectively, in Pan4. IGHV3-5 was the third most represented in Pan4 with 49 (1.07%) sequences. The number of different IMGT clonotypes was again observed to be relatively proportional to the number of assigned sequences. However, IGHV3-9 showed less diversity with 42 (31.57%) distinct IMGT clonotypes compared to IGHV4-2 with 58 (43.60%) different clonotypes.

For IGHD, Pan0 contained a very diverse representation of IGHD genes (Figure 2B), with multiple genes for IGHD1 (8), IGHD2 (6), IGHD3 (4), IGHD4 (3), IGHD5 (3), and IGHD6 (6). In the case of IGHD7, only a single gene (IGHD7-1) was called, using 34 sequences assigned to this clonotype. With this exception, all other IGHD families were represented with a higher number of assigned sequences (from 327 to 1,331) with clonotype analyses. The number of assigned IMGT clonotypes was also highly diverse in regard to clonotypes called, with the most abundant genes having the highest numbers of IMGT clonotypes. The results obtained from Pan4 were strikingly different. The IMGT clonotype assignment of IGHD sequences was almost exclusively dominated by IGHD2-2, which was comprised of 3,948 (85.93%) sequences. IGHD6-1 and IGHD4-2 were the next most abundant genes present with only 2.72 and 2.31% of the assigned sequences, respectively. Despite the enriched representation in regards to sequence number, the IGHD2-2 clonotypes seen were not particularly diverse, encompassing only 22 (16.29%) of the detected differential IMGT clonotypes, while IGHD6-1 and IGHD4-2 were represented by 11 (8.15%) and 8 (5.92%) clonotypes, respectively. Surprisingly, IGHD3-3, which had only 67 (1.45%) assigned sequences, included the most variable selection of IMGT clonotypes at 23 (17%).

Similarly, a diverse collection of IGHJ genes was observed in Pan0 (Figure 2C). IGHJ4 was the most abundantly represented with 2,491 (47.57%) sequences. The next most abundant were IGJ5-1 and IGHJ6 with 1,108 (21.16%) and 891 (17%) sequences, respectively. As was observed for prior Pan0 analysis, the number of different IMGT clonotypes was proportional to the most represented genes. However, in regard to Pan4, IGHJ genes were again highly enriched for the IGHJ5-1 gene, represented by 3,978 (86.59%) of the assigned sequences while IGHJ4 was only a minor group with 433 (9.42%) assigned sequences. Overall, the variety of clonotypes seen was generally low, but the most represented genes included the majority of different clonotypes.

**Figure 3:**
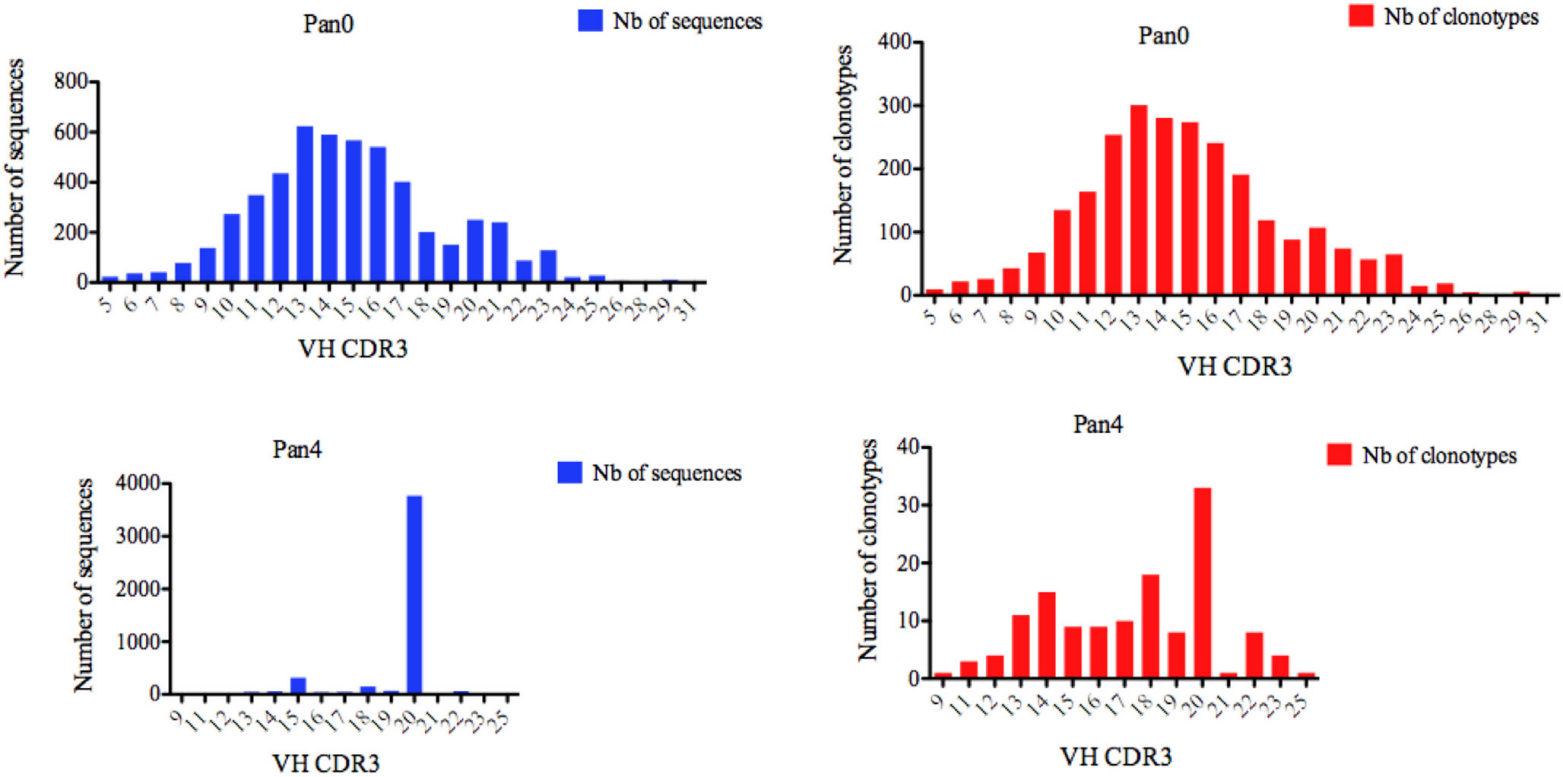
Number of IGH sequences and clonotypes distributed by VH CDR3 length. VH CDR3-IMGT length was determined by IMGT/HighV-QUEST in productive (those with in-frame junctions, C104, W118) scFv sequences. The numbers of clonotypes per VH CDR3 length are plotted in Pan0 and Pan4 libraries. Nb: number.

We next turned our attention to VH CDR3 lengths, as these have been previously correlated with anti-HIV antibody activity. A variety of VH CDR3 lengths were observed in the Pan0 library, ranging from 5 AA and up to 31 AA-long (Figure 3). Within these VH, 78.29% had a CDR3 size of 17 AA or less, and 82.14% had a CDR3 of 18 AA or less. A similar trend was observed with the number of IMGT clonotypes, where the most prevalent VH CDR3 lengths were represented by the most diverse pool of clonotypes. In the Pan4 libraries, the VH CDR3 length extended from 9 to 25 AA, however, the vast majority of the assigned sequences (3,769, 82.04%) had a CDR3 of 20 AA. The next most abundant length observed was 15 AA with 316 (6.88%) assigned sequences, followed by 18 AA with 148 (3.22%). The numbers of different clonotypes contained within these VH CDR3 size bins were usually low. For example, the 20 AA-long VH CDR3, which represented 82.04% of the total assigned sequences, showed relatively poor diversity with only 33 (24%) of the different IMGT clonotypes represented. This was followed by the next most clonotypically diverse VH CDR3 length bins, including 15 AA CDR3 (11.11%) and 18 AA CDR3 (13.33%). Consequently, we observed a more diversified range of VH CDR3 lengths when considering clonotype distribution rather than the simple number of sequences (Figure 3) per VH CDR3 AA length.

#### IGKV

**Figure 4:**
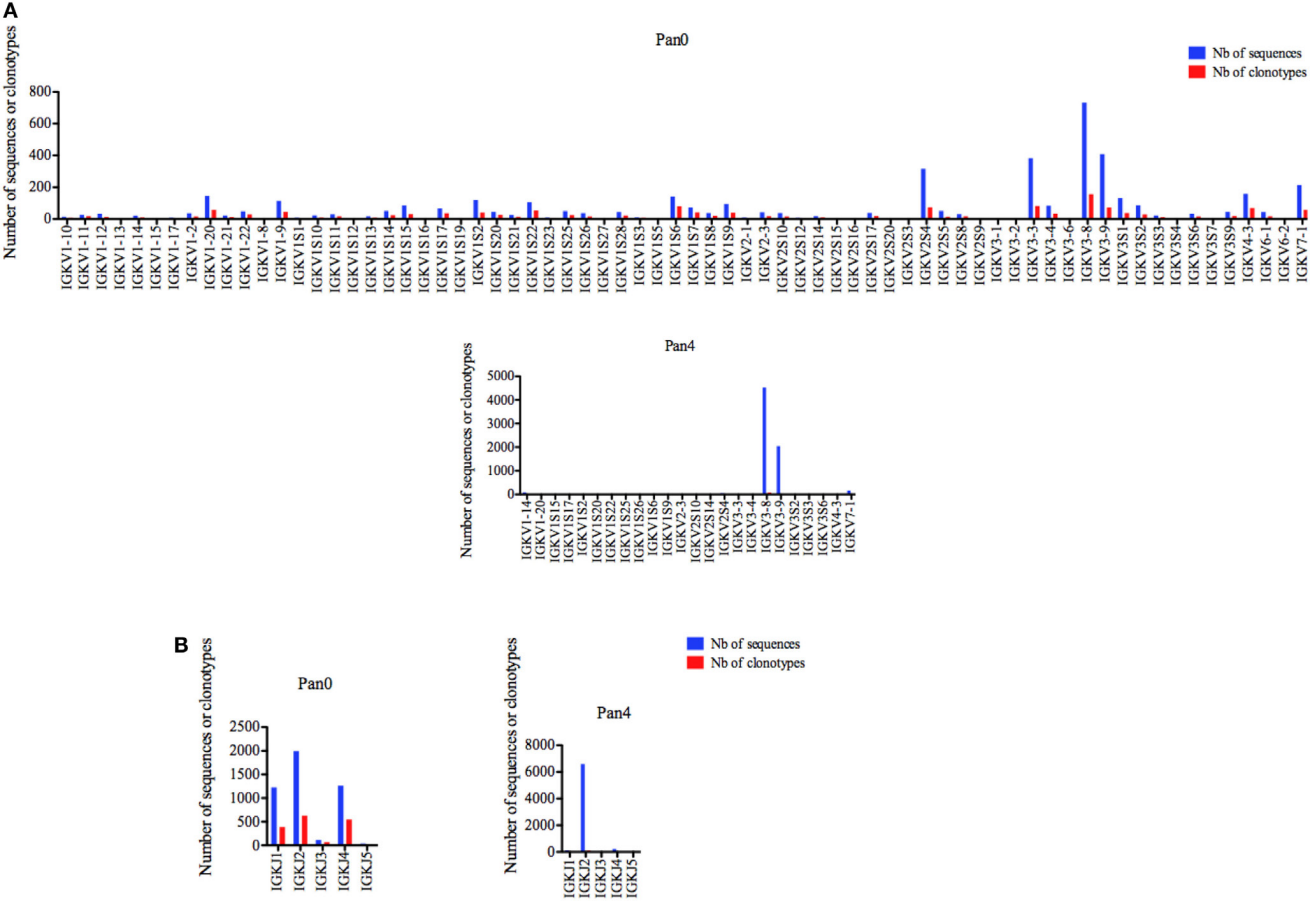
Number of IGK sequences and clonotypes, per IGKV and IGKJ gene, in Pan0 and Pan4 scFv libraries. An IMGT IGKV clonotype (AA) was defined as a unique V-J rearrangement using IMGT gene and allele names as determined by IMGT/HighV-QUEST at the nucleotide level, and a unique, in frame V-KAPPA CDR3-IMGT AA junction (C104, F118) (56). **(A)**, IGKV; **(B)**, IGKJ. Nb: number.

A diversified representation of IGKV genes was observed within Pan0, which identified 69 unique IGKV genes (Figure 4A). The most abundantly represented were IGKV3-8, IGKV3-9, IGKV3-3, and IGKV2S4 with 732 (16.24%), 408 (9.05%), 383 (8.50%), and 316 (7.01%) sequences assigned, respectively. As previously observed with IGHV, the number of different IMGT clonotypes was directly proportional to the gene abundance described above. A less diverse pool of 23 IGKV genes was obtained from the Pan4 data. These genes were dominated by the IGKV3-8 and IGKV3-9 genes with 4,525 (65.04%) and 2,043 (29.37%) of assigned sequences, respectively. The next most abundant gene characterized was IGKV7-1 with 163 (2.34%) assigned sequences. The number of different clonotypes was proportional and dominated by IGKV7-1 and IGKV1-14 with 82 (55.78%) and 16 (10.88%), respectively; IGKV7-1 contained 13 (8.84%) different clonotypes.

Five different IGKJ genes (1–5) were detected in Pan0 (Figure 4B), with the most abundant being IGKJ2, IGKJ4, and IGKJ1 with 1,993 (43.03%), 1,263 (27.26%), and 1,227 (26.49%), respectively. The number of different clonotypes observed was also highest for IGKJ2, IGKJ4, and IGKJ1 genes with 629 (38.21%), 549 (33.35%), and 390 (23.69%), respectively. For Pan4, 4 IGKJ genes were detected (Figure 4B), with IGKJ2 being the most abundant with 6,596 (94.83%) assigned sequences. A similar abundance was observed for the number of different IMGT clonotypes within IGKJ2, which was shown to have 102 (69.86%) assigned clonotypes.

**Figure 5:**
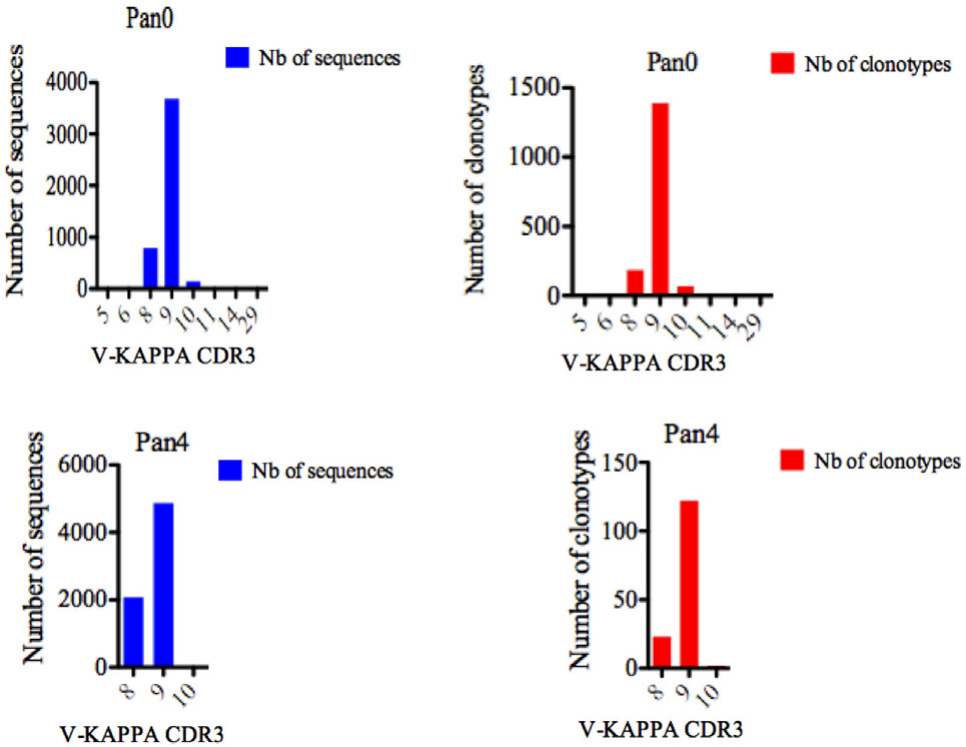
Number of IGK sequences and clonotypes, distributed by V-KAPPA CDR3 length. V-KAPPA CDR3-IMGT length was determined by IMGT/HighV-QUEST in productive (those with in-frame junctions, C104, F118) single chain fragment variable sequences. The numbers of clonotypes per V-KAPPA CDR3 length are plotted in Pan0 and Pan4 libraries. Nb: number.

For Pan0, V-KAPPA CDR3 lengths ranged from 5 AA to 29 AA long (Figure 5), with the most represented lengths being 9 and 8 AA, comprising 3,690 (79.52%) and 794 (17.11%) assigned sequences, respectively. A similar relationship was observed for the number of different IMGT clonotypes within these V-KAPPA CDR3 length bins, with 1,390 (29.95%) and 185 (11.2%) for 9 and 8 AA-long CDR3, respectively. In Pan4, the observed V-KAPPA CDR3 were between 8 and 10 with 9 and 8 AA-long CDR3 lengths dominating the pool as both the most represented in sequence space, as well as the most diverse, containing 122 (83.00%) and 23 (15.64) different clonotypes, respectively.

#### IGLV

**Figure 6:**
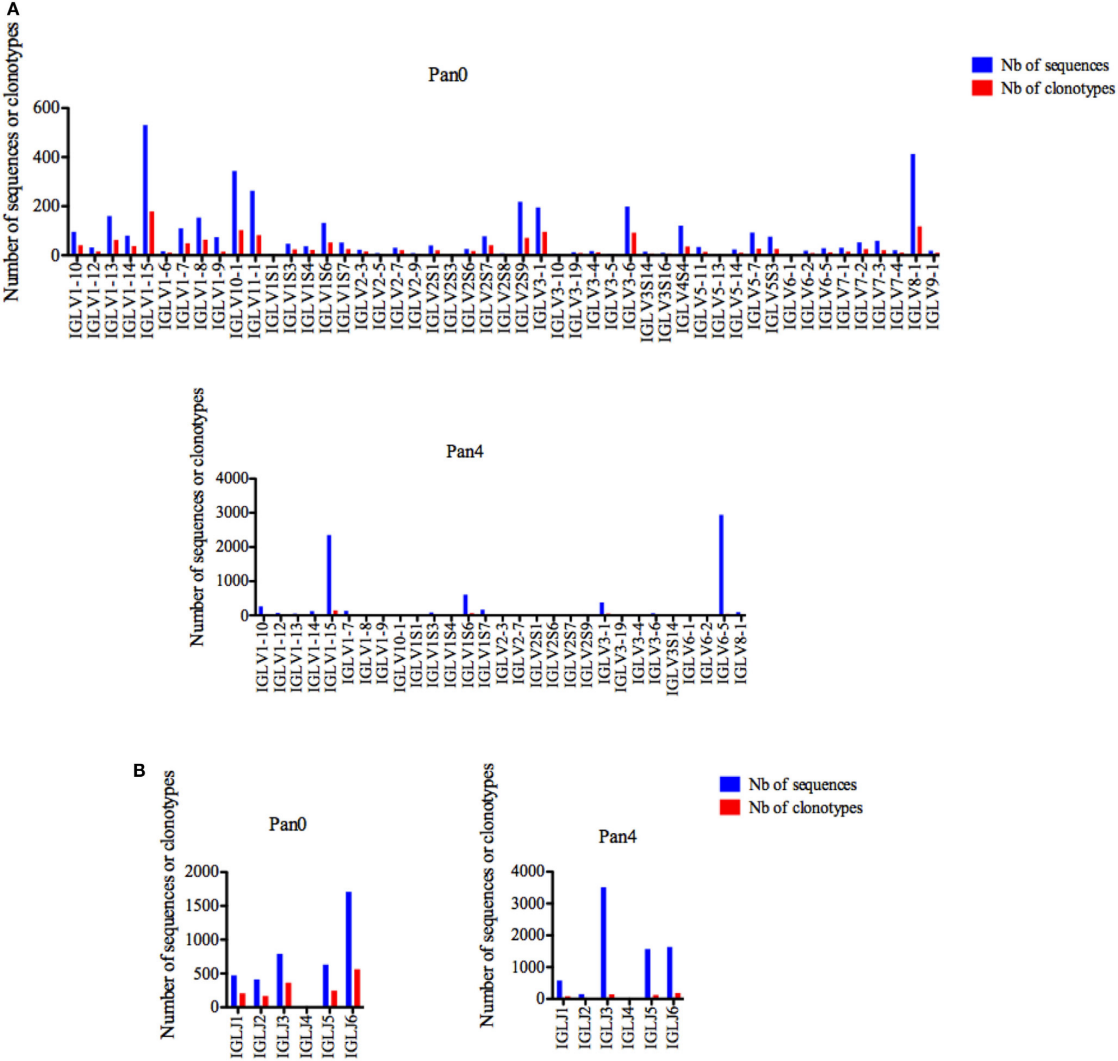
Number of IGL sequences and clonotypes, per IGLV and IGLJ gene, in Pan0 and Pan4 single chain fragment variable libraries. An International ImMunoGeneTics information system (IMGT) IGLV clonotype (AA) was defined as a unique V-J rearrangement using IMGT gene and allele names as determined by IMGT/HighV-QUEST at the nucleotide level, and a unique, in frame V-LAMBDA CDR3-IMGT AA junction (C104, F118) (56). **(A)**, IGLV; **(B)**, IGLJ. Nb: number.

Similar to IGKV, IGLV gene usage was also shown to be highly diverse in the Pan0 library, including 48 unique IGLV genes (Figure 6A). The most abundant genes were IGLV1-15, IGL8-1, and IGLV10-1 with 531 (13.15%), 413 (10.23%), and 344 (8.52%) sequences, respectively, and the number of clonotypes was proportional to that of the observed gene abundances. However, in the Pan4 sub-library only 28 different IGLV genes were observed (Figure 6A). IGLV1-15 and IGLV6-5 were the most abundant. IGLV1-15, the most represented gene in Pan0, was further enriched in Pan4 with 2,350 (31.44%) assigned sequences. IGLV6-5, which represented only 0.71% of the scFv from the Pan0 library, was the most abundant sequence observed in Pan4 with 2,939 (39.33%) of the assigned sequences. As seen within the other IG genes, the number of clonotypes was proportional to the relative representation of each gene in the pool.

Six different IGLJ genes were identified within the Pan0 library (Figure 6B). IGLJ6 and IGLJ7 were the most dominant, with 1,708 (42.49%) and 793 (19.73%) sequences assigned, respectively. In the Pan4 library, 5 different IGLJ genes were observed, missing only the presence of the IGLJ4 gene as compared to the Pan0 library. Despite this similar level of diversity, the majority of sequences were IGLJ3 and IGLJ6 with 3,511 (47.09%) and 1,636 (21.94%), respectively. Once again, the number of clonotypes was again proportional to the most represented genes.

**Figure 7:**
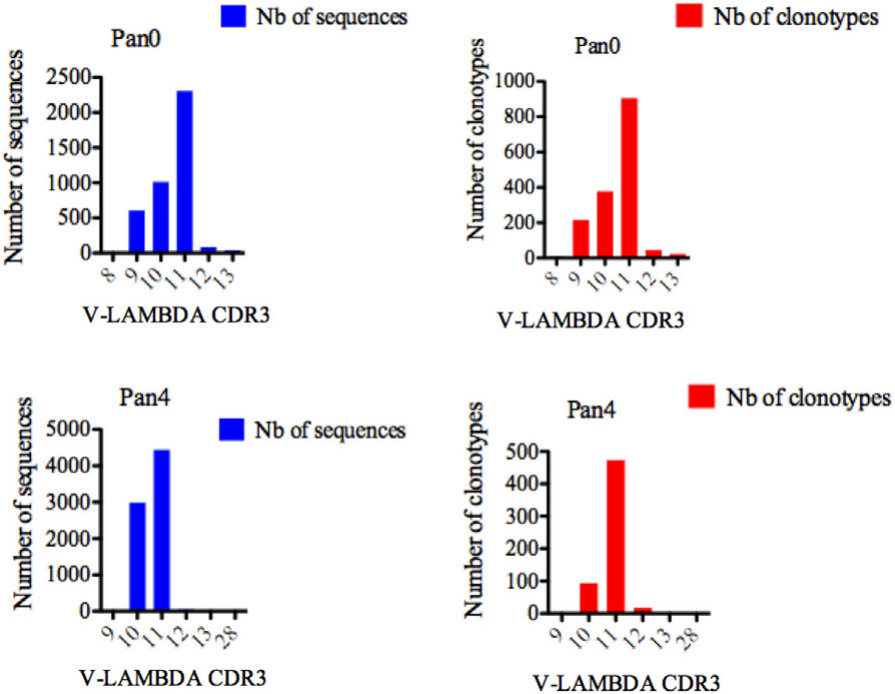
Number of IGL sequences and clonotypes, distributed by V-LAMBDA CDR3 length. V-LAMBDA CDR3-IMGT length was determined by IMGT/HighV-QUEST in productive (those with in-frame junctions, C104, F118) single chain fragment variable sequences. The numbers of clonotypes per V-LAMBDA CDR3 length are plotted in Pan0 and Pan4 libraries. Nb: number.

Single chain fragment variable from the Pan0 library contained V-LAMBDA CDR3 lengths ranging from 8 to 13 AA (Figure 7), with the majority of CDR3 lengths in the pool comprised of 11 (2,308 sequences, 56.73%), 10 (1,013 sequences, 24.90%), and 9 (608 sequences, 14.94%) AA. V-LAMBDA CDR3 lengths within the Pan4 library were observed to vary from 9 to 28 AA, but were dominated by CDR3 lengths of 11 and 10 AA, representing 4,443 (59.36%) and 2,988 (39.92%) of the assigned sequences, respectively. In both Pan0 and Pan4, the overall number of clonotypes was proportional to the assigned genes sequences; specifically in Pan4 the 11 AA V-LAMBDA CDR3 group contained the highest number of diverse clonotypes, followed by those with 10 AA CDR3, with 473 (80.44%) and 93 (15.81%) respectively.

#### VH-VL Combinations

A clear advantage of the SMRT sequencing over other NGS approaches is the generation of longer reads, which for phage display libraries should allow the analysis of full length scFv and identification and interrogation of productive VH-VL combinations. To address this, the IMGT/HighV-QUEST output data were manually inspected to identify VL combinations for the IGHV clonotypes corresponding to the two recovered clones. The S1 VH was associated with three productive IGVL genes, including two sequences from IGLV1-15 and one from IGLV1-10. The S2 VH was associated with 102 productive VL sequences, including 76 IGLV2S1, 8 IGLV6-5, 5 IGKV3-8, 3 IGLV1-15, 2 IGLV1S6, and 1 sequence assigned to other genes (IGKV3-9, IGLV1-10, IGLV1-15, IGLV1-7, IGLV1S3, IGLV2-3, IGLV2-7, and IGLV6-1).

#### Batch Analysis of Triplicate SMRT Cell Outputs

When screening a highly variable library, a limitation of the PacBio RSII system is the relatively low read depth per SMRT cell, as compared to other NGS technologies. We observed this when running single SMRT cells per sample in our experiments. However, this can be countered by running multiple SMRT cells per sample to increase read depth and the ability to detect low level variants within a population. As the IMGT statistical and clonotype analysis tool functions with batched data, including multiple SMRTcells (up to one million sequences), we performed a batch analysis for triplicate, pooled samples from Pan0 and Pan4 (Table 1). In this batched dataset, for IGHV, the number of sequences assigned to an IMGT clonotype was increased (two- to fourfold) for Pan0 and almost threefold in Pan4 as compared to those sequences generated with a single SMRT cell per sample (Pan0_1, Pan0_2, and Pan0_3 or Pan4_1, Pan4_2, and Pan4_3). The number of IMGT clonotypes was also approximately twofold of that called using single SMRT cell data for both Pan0 and Pan4. Similar increases in the number of sequences and clonotypes were also observed for IGKV and IGLV when combining outputs from triplicate SMRT cells for both Pan0 and Pan4 (Table 1). These observations highlight the need to consider increasing depth of coverage when screening highly variable libraries using single molecule sequencing approaches, either through batching multiple cells of data or generating sequence data using a higher throughput instrument (i.e., PacBio Sequel system).

## Discussion

Understanding the role of polyclonal nnAbs in the inhibition of HIV-1 infection will require large-scale analyses of virus-specific antibodies. These analyses should evaluate the functionality of the antibodies, as well as characterize their structure, including V-(D)-J gene or allele usage and CDR3 lengths. Techniques that rely on antigen-based selection of single B cells or screening of single colonies following phage display generally result in a low number of sequences and are not always suited to examine the expected level of complexity in such a system. Broadly used NGS technologies, such as Illumina, can generate very high numbers of sequence reads, but are limited in read length and can cover no more than one variable domain (VH or VL) per read, limiting data interpretation and the ability to evaluate the impact of specific VH–VL pairing.

Here, we evaluated the ability of SMRT sequencing technology to generate full length scFv sequencing reads for the analysis of phage display libraries. We obtained thousands of highly accurate (≥99.9%) sequence reads, each containing productive VH-VL combinations. As previously reported for this rhesus macaque (11), SIV Env gp140-specific scFv were dominated by the usage of IGHV4-2 and IGHV3-9. However, we observed far fewer IGHV1-1-containing scFvs in the Pan4 library than expected. We also observed more IGHV3-9-contaning scFv sequences than those with IGHV4-2, despite the latter being more abundant in the unpanned library. Together, these data suggest that the SMRT sequencing approach will be useful for the characterization of scFv libraries as more comprehensive rhesus V gene databases are being developed (60, 61).

The libraries examined were very diverse and included a great number of distinct clonotypes that were, in general, proportional to the number of sequences obtained for each gene. Interestingly, SIV Env gp140-specific scFvs were characterized by a more limited number of clonotypes, as has been previously reported for both HIV-1 and SIV antibodies (12, 22, 23, 32, 62). Specifically, the most dramatic examples were observed with IGHD and IGHJ genes, in which more than 85% of sequences contained IGHD2-2 and IGHJ5-1. It will be necessary and informative to determine whether these patterns are similar in other experimentally infected animals, particularly in those immunized with SIVmac239Δnef, which show protection from SIVmac239 challenge infection. Though less pronounced in terms of clonotype distribution, SIV Env gp140-specific scFvs presented an average of VH CDR3 size of 20 AA (representing around 80% of total sequences), which was far greater than that seen in the Pan0 library, containing the same proportion of sequences for VH CDR3 ranging between 5 and 17 AA. It is difficult to interpret the relevance of these results to nnAbs in particular, as the plasma of this rhesus macaque also contained a high titer of neutralizing antibodies. Understanding the impact of VH CDR3 length on antibody functionality and disease outcome will definitely require a similar analysis across a greater number of animals.

Additional high-quality sequences were obtained for V-LAMBDA antibodies containing a diversified pool of genes from the major IGLV subgroups. IGVL genes from SIV Env gp140-specific scFvs were generally derived from among the most represented genes in the Pan0 library, with the exception of IGLV6-5, which showed a ~40-fold increase in representation in the SIV Env gp140-specific scFv library, compared to that from the total, unpanned IG repertoire. Due to the combinatory nature of VH-VL association of our phage display libraries, further analyses will certainly be required to clarify and understand the potential contribution of diverse IGKV and IGLV genes to the overall antibody functionality.

It has also been observed that the IGHV1 (34.4%) and IGHV4 (26.15%) subgroups were the most dominant among naïve IgM libraries in human adults but that these representations could be altered during early (40 days) or advanced (8 months) HIV-1 infection with differences between IgM and IgG frequencies, as well as between peripheral blood mononuclear cells (PBMC) and bone marrow (BM) compartments (63). During early HIV-1 infection, IGHV1 and IGHV3 (respectively, 40.96% in PBMC and 51.22% in BM IgM libraries, and, respectively, 35.13% in BM and 33.8% in PBMC IgG libraries) were the most dominant while IGHV3 and IGHV4 (respectively, 38.42% in PBMC and 23.69% in BM IgM libraries), and specifically IGHV4 in IgG libraries (PBMC 27.89% and BM 28.32%) were the second most dominant (63). During the more advanced (8 month) stage of infection, IGHV3 (57.10% in PBMC, 59.31% in BM IgM libraries) and IGHV1 (53.03% in PBMC and 46.29% in BM IgG libraries) were the most dominant while IGHV4 and IGHV1 (respectively, 17.18% in PBMC and 15.98% in BM IgM libraries), and IGHV3 (22.96% in PBMC, 29.15% in BM IgG libraries) were the second most dominant (63). The complexity of these data suggest that analyses of multiple animals and infection time points are needed, as well as a comparative analyses of these data compared to those generated using scFv libraries constructed with rhesus specific-primers to ensure that any rhesus-specific IG populations are included (9, 12). These additions, as well as leveraging the growing rhesus databases (60, 61) will be necessary moving forward to ensure the most comprehensive analysis of polyclonal antibody responses during SIV infection in rhesus macaques.

There are several caveats that are worth noting regarding the approaches used to generate the V-(D)-J composition and CDR3 lengths of SIV-specific antibodies identified and characterized in this study.

The first caveat surrounds the relatively low read depth generated by the SMRT sequencing approach. As mentioned earlier in the text, He et al. generated over one million raw reads (750–950 bp) using the PGM-S5 system in a similar study investigating HIV-specific scFv antibodies. Interestingly, these data demonstrated similar sequence output size from the IMGT statistical and clonotype analysis. Hemadou et al. analyzed more than 450,000 reads using a SMRT sequencing strategy, but by combining the data from 15 SMRT cells to increase depth. In the current work, we did observe an increase (two- to fourfold) of individual sequences or clonotypes called when 3 SMRT cells of data were combined prior to clonotype analysis. While the current data are already hypothesis generating, the increased information gained by increasing read depth underscores the need for future, similar studies to batch data from multiple RSII SMRT cells or to move this type of study to the higher throughput Sequel system, which generates 400,000–500,000 single molecule reads per chip, drastically increasing the single molecule read depth available.

The next caveat relates to our use of human-based primers for the construction of phage display library under examination. Overall, the rhesus macaque genome is highly homologous to that of the human (~93%) (64). In particular, Sundling et al. found a high level of homology (~92%) between the human and macaque functional VH regions, with genes clustered according to family distribution rather than species when examined by phylogenetic analysis (65). However, it is noted that these authors also identified unique macaque-specific antibody gene families. Consequently, the phage display library used in the current study is likely biased toward gene families with high homology to the corresponding human genes. It is the focus of our immediate future work to recapitulate these data and expand upon them with the use of macaque-specific primers (9, 12, 62, 65–67). For example, Dai et al. observed a diverse repertoire of VH CDR3 length by tracking lineages of CD4-binding site-directed mAbs in macaques immunized with an HIV-1 trimer vaccine (62). Our VH CDR3 results differed from these observations, which may be due to this type of primer bias, or due to the fact that our antibodies are targeting the MPER region of Env, rather than the receptor-binding site. In fact, our variable light chain results were comparable to those of Dai et al., where it was observed that the 1 or 2 CDR3 AA length was most abundant for V-KAPPA CDR3 and V-LAMBDA CDR3. A related caveat pertains generally to the characterization of all combinatorial phage display approaches, mainly that our libraries do not represent authentic VH–VL pairing. Methods to clone paired VH-VL regions from single macaque B cells have been developed (9, 66). These studies relied on Sanger sequencing while other macaque-based studies did not apply NGS to characterize authentic VH–VL pairs (62, 65). Moving forward, the use of combinatorial phage display technology should be complemented with single cell-based methodologies, including droplet emulsion-based approaches, to resolve relevant VH–VL pairing (68–72).

Another caveat that could impact our V-(D)-J characterization of SIV gp140-specific antibodies was the use of the IMGT database for classification and analyses, as it possesses an incomplete representation of macaque IG germline sequences. In their study Dai et al. address this concern by comparing antibody V-(D)-J compositions obtained *via* their in-house “CS germline-gene database” to that of the IMGT database, observing a more diversified VH family composition (mostly VH1, VH2, and VH3) using their in-house tool, while IMGT-generated data were skewed toward VH1 and VH4. Although we exclusively used IMGT analyses in this study, we also found that VH3 and VH4 family genes were among the most represented within the SIV gp140-specific antibodies. These two families were followed by VH1 albeit to a lesser level than what was observed by Dai et al. or our previous study (11). It is possible that the differences between our results and those from Dai et al. were individual specific, or due to the varied panning antigens used. Using Sanger sequencing, we have previously observed VH4-skewed usage in gp120-specific antibodies from the same animal examined in this current study (11). Most importantly, we are currently working with IMGT to expand their macaque IG germline database, as to eliminate this caveat in future studies. To note, Dai et al. also observed similar patterns of VH CDR3 lengths and SHM levels between IMGT and their own “CS germline-gene database” results. Here, we also observed a wide range of VH CDR3 lengths in the context of differential clonotype identities, which is probably more relevant for functional antibody classification.

Lastly, engineering of native-like SIV Env scaffolds may be necessary for the identification of highly functional antibodies, particularly those with potent neutralization activity. For instance, we have previously demonstrated the relative stability of non-engineered soluble SIVmac239 Env gp140 trimer and its ability to deplete SIV-infected macaque plasma of antibodies with neutralization activity against the neutralization sensitive SIVmac316 isolate (20). The depletion was less effective against neutralization resistant SIVmac239, although this was expected as the plasma did not display much neutralization activity against SIVmac239 in the first place (20). Subsequent follow up proved challenging, as we were unable to isolate potent SIV neutralizing antibodies although the phage display library had been constructed from an SIV-infected macaque with unusually high neutralization titers (11). This failure may have be due to the use of soluble SIVmac239 Env gp140 trimer, which likely would be improved through specific engineering as has been described for HIV-1 (22, 23, 30, 31, 33, 73).

## Author Contributions

IF, RS, MLS, and M-PL designed the study and wrote the manuscript. IF, SH, DH, BC, WC, and MS performed scFv DNA preparation. AA and GD performed PacBio the SMRT sequencing. IF performed scFv recovery from sublibrary. SK, PD, and M-PL developed and assisted with the IMGT analysis tools and web portal (www.imgt.org). All other authors have read and approved the final manuscript.

## Conflict of Interest Statement

The other authors declare that the research was conducted in the absence of any commercial or financial relationships that could be construed as a potential conflict of interest.

## Abbreviations

SIV, simian immunodeficiency virus; Env, envelope; scFv, single chain fragment variable; IMGT®, the international ImMunoGeneTics information system; bp, base pair; IG, immunoglobulin; CDR, complementarity determining region; AA, amino acid.
